# The Calcium-Sensing Receptor and the Parathyroid: Past, Present, Future

**DOI:** 10.3389/fphys.2016.00563

**Published:** 2016-12-15

**Authors:** Arthur D. Conigrave

**Affiliations:** Faculties of Science and Medicine, School of Life and Environmental Sciences, Charles Perkins Centre, University of SydneySydney, NSW, Australia

**Keywords:** calcium-sensing receptor, parathyroid, phospholipase C, adenylate cyclase, heterotrimeric G proteins, Calcimimetics, calcilytics, mineral metabolism

## Abstract

Parathyroid hormone (PTH) defends the extracellular fluid from hypocalcemia and has powerful and well-documented actions on the skeleton and renal tubular system. To achieve a satisfactory stable plasma calcium level, the secretion of PTH, and the resulting serum PTH level, is titrated carefully to the prevailing plasma ionized Ca^2+^ concentration via a Ca^2+^ sensing mechanism that mediates feedback inhibition of PTH secretion. Herein, I consider the properties of the parathyroid Ca^2+^ sensing mechanism, the identity of the Ca^2+^ sensor, the intracellular biochemical mechanisms that it controls, the manner of its integration with other components of the PTH secretion control mechanism, and its modulation by other nutrients. Together the well-established, recently elucidated, and yet-to-be discovered elements of the story constitute the past, present, and future of the parathyroid and its calcium-sensing receptor (CaSR).

## Introduction

The parathyroid gland elaborates a peptide hormone, parathyroid hormone (PTH) whose primary role is to prevent and/or reverse acute hypocalcemia. It achieves this by: mobilizing calcium from stores in bone; stimulating renal Ca^2+^ reabsorption; and promoting the production of 1,25-dihydroxyvitamin D_3_ to drive intestinal calcium absorption. To prevent uncontrolled elevations in plasma calcium concentration in response to PTH, a molecular feedback mechanism mediated by the extracellular Ca^2+^ ion concentration (Ca^2+^_o_) suppresses PTH secretion from the cells of the gland (review: Conigrave and Ward, 2013). While this mechanism operates primarily on parathyroid chief cells, which are the most numerous cell type and major site of PTH production, it may also operate on a second less numerous cell type, the parathyroid oxyphil cells (Ritter et al., 2012). In addition to providing acute control of PTH secretion from both newly-formed secretory vesicles and stored secretory granules, the Ca^2+^-mediated feedback mechanism also suppresses the transcription of the PreProPTH gene and cell proliferation (review: Brown and MacLeod, 2001). Herein, I provide an account of how the pivotal parathyroid Ca^2+^ sensing mechanism was first characterized and how key biochemical features of the signaling mechanisms were exploited to clone the class C G-protein coupled receptor (GPCR) we now know as the calcium-sensing receptor (CaSR). I go on to describe how studies of this receptor in these cells have led to deep understandings of parathyroid function in health and disease and new approaches to therapies for various disorders of calcium metabolism and parathyroid function.

## The past

### *In vivo* and *in vitro* evidence for a parathyroid Ca^2+^ sensing mechanism

Surgical removal of the parathyroid glands, whether intentional or inadvertent, induces acute, and in some cases catastrophic, hypocalcemia in experimental animals and in humans (e.g., MacCallum and Voegtlin, 1909; MacCallum et al., 1914; Westerdahl et al., 2000; Vasher et al., 2010; Salinger and Moore, 2013). In addition, perturbations of the plasma ionized calcium concentration *in vivo* by intravenous infusions of calcium salts to induce hypercalcemia or Ca^2+^ chelators such as citrate or EGTA to induce hypocalcemia provoke rapid negative and positive changes in the serum PTH concentration respectively (Fox and Heath, 1981; Conlin et al., 1989; Schwarz et al., 1992). These studies demonstrate that the gland is equipped with a Ca^2+^-sensor that suppresses PTH secretion in response to elevated Ca^2+^ concentration.

The successful preparation of bovine parathyroid cells using collagenase digestion of sliced parathyroid gland tissue provided novel opportunities to assess the cellular Ca^2+^ sensing mechanism *in vitro* (Brown et al., 1976) and similar observations were made for porcine (Morrissey and Cohn, 1978) and also human (Birnbaumer et al., 1977; Brown et al., 1978a, 1979a; Conigrave et al., 2004) parathyroid cells. In all these cases, mammalian parathyroid cells in primary culture supported a robust endogenous secretion of PTH that was promptly shut off upon elevation of Ca^2+^_o_. In cells prepared from samples of parathyroid tissue derived from patients with primary hyperparathyroidism there was impairment but not complete loss of Ca^2+^_o_ sensitivity (Brown et al., 1979a,c; Mun et al., 2009). The behavior raises questions about the nature of the extracellular Ca^2+^ sensor. It also raises questions about the nature of the intrinsic/endogenous PTH secretion mechanism.

In the first description of a viable, functional parathyroid cell preparation (Brown et al., 1976) bovine parathyroid cells in primary culture in Eagle's medium (minus bicarbonate) secreted PTH linearly at a rate of 20–30 pmol cell^−1^ h^−1^ for up to 3 h. PTH secretion was suppressed by around 60% at a Ca^2+^_o_ of 1.5 mM when compared to that observed at 0.5 mM Ca^2+^_o_. In the presence of 0.5 mM Ca^2+^_o_, elevated extracellular Mg^2+^ concentration (Mg^2+^_o_) also suppressed PTH secretion although Mg^2+^_o_ was less potent than Ca^2+^_o_. Finally, increases in PTH secretion were observed in response to the β-adrenergic agonist isoproterenol that were partially reversed by the β-adrenergic antagonist propranolol (Brown et al., 1976). Thus, key features of the preparation included: Ca^2+^_o_- and Mg^2+^_o_-mediated suppression of PTH secretion, pointing to the existence of an intrinsic divalent cation sensor with a preference for Ca^2+^_o_ over Mg^2+^_o_; and stimulation of PTH secretion by cAMP-linked GPCRs including beta-adrenergic, dopaminergic, and prostanoid receptors (Brown et al., 1977a,b; Gardner et al., 1980). These findings pointed to the existence of neuronal, hormonal, and/or local stimulatory control of PTH secretion. Although not clearly identified, the findings also demonstrated the existence of an intrinsic PTH secretion mechanism. According to one interpretation, parathyroid cells are equipped with a constitutive PTH secretion mechanism. According to an alternative interpretation, parathyroid cells respond to an autocrine/paracrine mechanism that supports PTH secretion.

### The concept of a calciostat and an extracellular Ca^2+^ set-point

**Figure 1:**
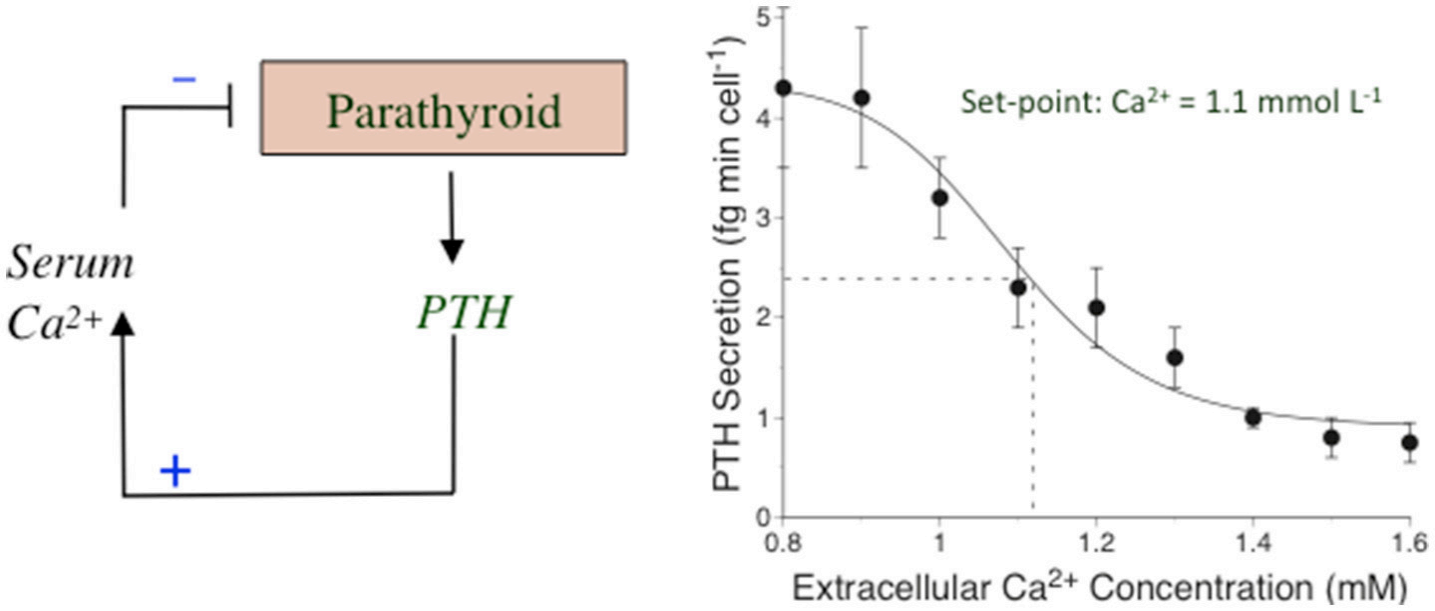
**The calciostat in parathyroid cells. Left:** A representation of the feedback mechanism by which PTH elevates the serum^*^ Ca^2+^ concentration and Ca^2+^ feeds back on the parathyroid to suppress PTH secretion in a process mediated by the CaSR. **Right:** Human parathyroid cells were perifused with HEPES-buffered physiological saline solutions containing various Ca^2+^ concentrations and samples of perifusate were collected at various times and subsequently analyzed for PTH1–84 as described in Conigrave et al. (2004). The results have been re-drawn. ^*^Total and ionized calcium concentrations are comparable in serum and plasma since the major calcium-binding protein, albumin is present in similar concentrations in both these fluids.

The Ca^2+^-sensing mechanism in the parathyroid supports the operation of an extracellular “calciostat” *in vivo*. The set-point for this calciostat occurs at a plasma ionized Ca^2+^ concentration of around 1.1–1.2 mM corresponding to plasma total calcium concentrations of around 2.2–2.4 mM, of which approximately half is in an albumin-bound form. PTH secretion rates rise 2 to 4-fold as Ca^2+^_o_ drops toward 1.0 mM and are effectively suppressed by >50% as Ca^2+^_o_ rises toward 1.4 mM (review: Conigrave et al., 2000a). The changes in PTH secretion rate are reflected in consonant changes in the serum PTH level (normal range 1–6 pmol/L). This set-point behavior can be readily demonstrated in perifused parathyroid cell preparations including those prepared from human parathyroid glands (Conigrave et al., 2004; Figure 1). Ca^2+^_o_-dependent inhibitory control of renal Ca^2+^ reabsorption, resulting in elevated renal calcium excretion, also contributes to the calciostat function, providing a key element of the defense against hypercalcemia (Kantham et al., 2009; Loupy et al., 2012).

### Extracellular Ca^2+^-mediated signaling mechanisms

#### cAMP promotes PTH secretion via a Ca^2+^-sensitive pathway

Suppression of cAMP levels accompanies high Ca^2+^_o_-induced suppression of PTH secretion in parathyroid cells stimulated to secrete by exogenous agonists of G_s_-coupled GPCRs (Brown et al., 1977a, 1979b, 1978b, 1985; Windeck et al., 1978) and also in cells not exposed to exogenous GPCR activators, in which intracellular cAMP levels are typically much lower (≤5% of those in stimulated cells; Brown et al., 1978b). Excellent correlations were observed between cAMP levels and PTH secretion rates in these experiments supporting the hypothesis that cAMP is a primary driver of both exogenous GPCR-stimulated and intrinsic PTH secretion (Brown et al., 1978b). Similar results were obtained in a comparative analysis of the effects of divalent and tervalent cations on PTH secretion and cAMP accumulation (Brown et al., 1990). If this is so, the mechanisms of Ca^2+^_o_-dependent suppression of cAMP levels and PTH secretion are different under the conditions of (i) exogenous, GPCR-stimulated and (ii) spontaneous PTH secretion. This follows because pertussis toxin disabled Ca^2+^_o_- and divalent/tervalent cation-induced suppression of dopamine-stimulated PTH secretion (Chen et al., 1989; Brown et al., 1990), demonstrating that G_i_ is required for inhibitory control of PTH secretion downstream of cAMP-linked GPCRs, but pertussis toxin had no dis-inhibitory effect on high Ca^2+^_o_-mediated suppression of intrinsic PTH secretion i.e., in the absence of exogenous GPCR activators (Brown et al., 1992). Findings in support of the hypothesis that pertussis toxin suppresses both exogenous GPCR-stimulated and endogenous PTH secretion (Fitzpatrick et al., 1986a) have not been confirmed.

**Figure 2:**
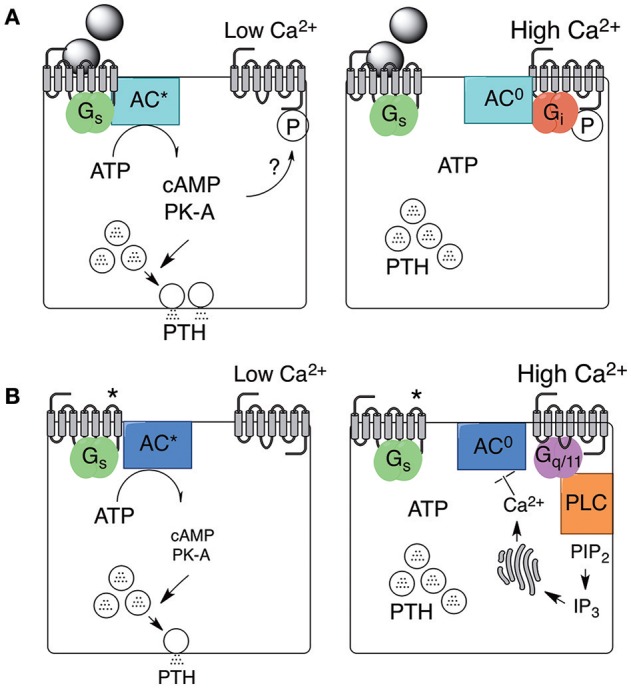
**Stimulated and spontaneous mechanisms in support of PTH secretion and its inhibition by high Ca^2+^_o_**. PTH secretion and its inhibition by high Ca^2+^_o_ arises from two distinct mechanisms. One mechanism is supported by exogenous agonists, including neurotransmitters or hormones, that activate G_s_-coupled GPCRs as shown in **(A)** (left and right). PTH secretion continues provided Ca^2+^_o_ remains low but is promptly inhibited by G_i_-dependent inhibition of adenylate cyclase in the presence of high Ca^2+^_o_. The mechanism by which the Ca^2+^_o_ sensor, now known to be the CaSR, preferentially binds to G_i_ in this context is not known but might depend on local protein kinase-A (PK-A) activation. A second mechanism occurs spontaneously and may be supported by constitutive G_s_-coupled GPCR activity (as shown in **B**; left and right) or by autocrine/paracrine production of receptor activators. PTH secretion via this second mechanism continues provided Ca^2+^_o_ remains low but is inhibited by high Ca^2+^_o_-induced G_q/11_-dependent activation of intracellular Ca^2+^ mobilization or Ca^2+^ influx (not shown). One possible mechanism by which increased intracellular free Ca^2+^ concentration (Ca^2+^_i_) suppresses PTH secretion is shown via Ca^2+^_i_-dependent inhibition of adenylate cyclase. ^*^Receptor activated in the absence of neuronal or hormonal stimuli.

The results suggest the existence of an extracellular Ca^2+^ sensor that is capable of activating G_i_ to suppress cAMP synthesis and, in turn, cAMP-linked PTH secretion in the presence of exogenous agonists that markedly elevate cAMP levels. The lack of association between G_i_, cAMP levels, and PTH secretion in parathyroid cells *NOT* exposed to exogenous GPCR activators, on the other hand, points to a distinct biochemical mechanism arising either from a second Ca^2+^ sensor or from a single Ca^2+^ sensor that couples to distinct downstream signaling pathways depending on whether the cells have been stimulated to secrete PTH by exogenous activators or are operating spontaneously (Figure 2). Support for the hypothesis that the Ca^2+^ sensing mechanism in parathyroid cells is mediated by Ca^2+^ channels and controlled by the activity of pertussis toxin-sensitive G-proteins (Fitzpatrick et al., 1986a,b) has not been supported by other studies (e.g., Brown et al., 1992). More recent work has implicated G_q/11_ and, possibly, phosphatidylinositol-specific phospholipase C (PI-PLC) and ERK_1/2_ downstream of an extracellular Ca^2+^ sensing GPCR (see below).

#### Intracellular Ca^2+^ mobilization and PI-PLC

An alternative signaling pathway, downstream of an extracellular Ca^2+^ sensor was subsequently identified in populations of bovine parathyroid cells loaded with the cell-permeant Ca^2+^-sensitive fluorophore fura-2AM. The cells exhibited robust intracellular Ca^2+^ transients in response to elevated Ca^2+^_o_ suggesting the action of a PI-PLC coupled GPCR that senses increases in Ca^2+^_o_ (Nemeth and Scarpa, 1986, 1987a). Furthermore, they exhibited similar intracellular Ca^2+^ transients in response to elevated Mg^2+^ or Sr^2+^ concentration consistent with the observations referred to above that the parathyroid Ca^2+^ sensing mechanism is promiscuous with respect to divalent cations (Chen et al., 1989; Brown et al., 1990). To investigate whether the parathyroid Ca^2+^ sensor might indeed be a PI-PLC coupled GPCR, further studies demonstrated that Ca^2+^, Mg^2+^ and other inorganic divalent cations promoted the production of water-soluble [^3^H]-inositol phosphates from [^3^H]-inositol labeled cells (Brown et al., 1987; Shoback et al., 1988).

### A promiscuous divalent/multivalent cation sensor

Investigation of the molecular requirements for divalent cation sensing in parathyroid cell preparations led to some surprising observations. Firstly, tervalent inorganic cations of the lanthanide group including Gd^3+^ and Tb^3+^ were found to be high potency activators (EC_50_ ≈ 5–50 μM) of parathyroid PI-PLC, suppressors of GPCR-stimulated cAMP accumulation, and inhibitors of PTH secretion (Brown et al., 1990) in a manner analogous to divalent cations. Furthermore, and even more surprisingly, organic multivalent cations including polyarginine, polylysine, and protamine (Brown et al., 1991a), the PLC inhibitor neomycin (Brown et al., 1991b), and polyamines such as spermine (Nemeth and Scarpa, 1987b) stimulated intracellular Ca^2+^ mobilization and inhibited PTH secretion.

### Expression cloning of a polyvalent cation-sensing receptor from a bovine parathyroid cDNA library

The demonstration that the parathyroid calcium sensor coupled to the activation of PI-PLC and, at least in certain circumstances, to heterotrimeric G_i_ G-proteins, and was promiscuous with respect to inorganic and organic multivalent cations provided a strategy by which a putative PLC-coupled receptor might be cloned by cellular expression of pools of mRNA derived from a size-fractionated bovine parathyroid cDNA library (Brown et al., 1993). Xenopus oocytes express a large conductance Cl^−^ channel whose open probability is highly sensitive to changes in intracellular Ca^2+^ concentration (e.g., downstream of GPCR-mediated generation of IP_3_ and intracellular Ca^2+^ mobilization). In this case, the successful cloning of the novel class C GPCR that is now referred to as “the calcium-sensing receptor” relied on its high degree of sensitivity to Gd^3+^, which was used to identify “active” pools of mRNA for further separation and purification. Once cloned, the receptor was readily expressed not only in Xenopus oocytes but also various mammalian cell lines including HEK-293 cells and was found to exhibit sensitivity not only to divalent inorganic cations including Ca^2+^ and Mg^2+^, and tervalent inorganic cations including Gd^3+^ but also to organic cations including the antibiotic neomycin (Brown et al., 1993), polyamines such as spermine (Quinn et al., 1997), cationic polypeptides such as polyarginine and polylysine (Ray and Northup, 2002), and cationic proteins including beta amyloid (Ye et al., 1997). The cloning of the bovine parathyroid CaSR was followed subsequently by the cloning of its orthologs from human parathyroid (Garrett et al., 1995), rat kidney (Riccardi et al., 1995), and rat brain (Ruat et al., 1995).

The CaSR is known now to be expressed widely, with various Ca^2+^_o_ dependent functions in cell and developmental biology as detailed elsewhere in this issue. It is also known to activate a large number of signaling pathways downstream of various G-proteins and multiple cell membrane-associated as well as cytoplasmic enzymes (review: Conigrave and Ward, 2013).

The CaSR mediates, for example, the activation of various protein kinases including protein kinase C isoforms, which negatively modulate CaSR function (Jiang et al., 2002; Davies et al., 2007; Lazarus et al., 2011; Young et al., 2014), and the mitogen activated protein (MAP) kinases ERK_1/2_, p38 and JNK (Kifor et al., 2001; Tfelt-Hansen et al., 2003; review: Conigrave and Ward, 2013). The roles of protein kinases in CaSR-mediated inhibitory control of PTH secretion are not well-understood but ERK_1/2_ appears to contribute (Corbetta et al., 2002) and could be activated downstream of either G_q/11_ or G_i_ (review: Conigrave and Ward, 2013).

While the CaSR is expressed and trafficked to the plasma membrane as functional homodimers (Bai et al., 1998, 1999) that couple efficiently to G_q/11_, it is also capable of forming heterodimers with other members of GPCR family C including metabotropic glutamate receptors (Gama et al., 2001) and GABA_B_ receptors, especially GABA_B1_ (Chang et al., 2007; Cheng et al., 2007). The consequences of heterodimerization for receptor localization to specific subdomains of the plasma membrane and for signaling pathway selection in different tissues and for the parathyroid, in particular, are not yet clear.

### Physiological and clinical significance of the CaSR for parathyroid function

#### Parathyroid and mineral disorders linked to CaSR mutations (and anti-CaSR antibodies)

As the bovine parathyroid, rat kidney, and human parathyroid CaSR cDNAs were cloned (Brown et al., 1993; Garrett et al., 1995; Riccardi et al., 1995), it became possible to assess whether any recognized human disorders of calcium metabolism and/or parathyroid function arose from mutations of the CaSR. This was rapidly confirmed for two hypercalcemic disorders in which the CaSR is hypofunctional: the uncommon disorder known as familial hypocalciuric hypercalcemia (FHH); and the extremely rare disorder known as neonatal severe hyperparathyroidism (NSHPT; Pollak et al., 1993, 1994; reviews: Brown et al., 1995; Hendy et al., 2000). It was subsequently also confirmed for the hypocalcemic disorder known as autosomal dominant hypocalcemia (ADH; Pearce et al., 1996) in which the CaSR is hyperfunctional.

##### FHH

Deactivating, typically heterozygous, mutations of the CaSR gene in FHH result in impaired or disabled Ca^2+^_o_-dependent inhibition of renal Ca^2+^ reabsorption, leading to hypocalciuria, and as well as impaired Ca^2+^_o_-dependent feedback inhibition of PTH secretion, typically without frank elevations in the serum PTH level as a result of associated increases in Ca^2+^_o_ (Chu et al., 1995; review: Brown et al., 1995). Instead, the set-point for Ca^2+^_o_-dependent suppression of PTH secretion rises thereby increasing the value of the calciostat and the steady-state Ca^2+^_o_ adopts this new level. The primary driver for the increase in Ca^2+^_o_ appears to be impaired renal calcium excretion, resulting in characteristic hypocalciuria (uCa/Cr ratio < 0.04 mmol mmol^−1^; uCa excretion < 1.5 mmol d^−1^). With the identification of two variants of FHH arising from mutations of two other genes, Gα11 and AP2S (see below), the major form of FHH that arises from mutations of the CaSR has been recently renamed FHH1.

##### NSHPT

In contrast to FHH, homozygous or compound heterozygous deactivating mutations of the CaSR gene have been linked to a severe hypercalcemic disorder that presents in neonatal life with total plasma calcium concentrations that may exceed 4.0 mM (Ward et al., 2004). In addition, there are marked elevations in the serum PTH level, indicative of near-total failure of Ca^2+^_o_-mediated feedback control of PTH secretion along with skeletal demineralization and pathological fractures (Pollak et al., 1993; review: Brown et al., 1995). The disorder responds promptly to total parathyroidectomy i.e., excision of all four parathyroid glands (Marx et al., 1986) demonstrating that the bone disease is driven by severe primary hyperparathyroidism.

Whether still more severe disorders of skeletal development and metabolism might arise from other types of CaSR mutations is not yet clear. Recently developed mouse models, however, suggest that this is so (Chang et al., 2008; Richard et al., 2010; reviews: Goltzman and Hendy, 2015; Santa Maria et al., 2016). An authoritative database of CaSR mutations and their links to human disease is maintained at: http://www.casrdb.mcgill.ca/.

##### ADH

Two other rare mineral disorders affecting the parathyroid arise from activating mutations of the CaSR. In one, autosomal dominant hypocalcemia, there is hypocalcemia and inappropriately normal or frankly low serum PTH levels arising from a reduction in the set-point for extracellular Ca^2+^ (Pearce et al., 1996). One or more of the following may also be observed: hypercalciuria, consistent with enhanced inhibition of renal Ca^2+^ reabsorption; hypocalciuria (e.g., Tan et al., 2003), consistent with reduced glomerular filtration of Ca^2+^ ions and a largely intact renal Ca^2+^ reabsorption mechanism; hypomagnesemia; and hyperphosphatemia (reviews: Thakker, 2004; Egbuna and Brown, 2008). This is typically a chronic benign condition, often diagnosed as an incidental finding on plasma biochemical analysis, in which there may be a longstanding history of paresthesiae, intermittent fasciculations and/or contractions of isolated muscle groups. There may also be a history of one or more childhood seizures including febrile convulsions (reviews: Thakker, 2004; Egbuna and Brown, 2008).

In a second disorder, arising from more severe activating mutations of the CaSR, a form of renal salt wasting also occurs. This Bartter Syndrome (type-5) arises from unrestrained CaSR activation on the contraluminal membrane of the thick ascending limb, which disables NKCC2-dependent NaCl reabsorption (reviews: Gamba and Friedman, 2009; Riccardi and Brown, 2010).

The impact of gene dosage on the severity of autosomal dominant hypocalcemia has been evaluated in a mouse model, the *Nuf* mouse (L723Q, affecting a residue at the C-terminal end of iL-2), which exhibits hypocalcemia, suppressed serum PTH levels, hypocalciuria, hyperphosphatemia, and ectopic mineralization and cataracts (Hough et al., 2004). All aspects of the phenotype were more severe in homozygous when compared to heterozygous mice demonstrating that a gene dosage effect applies in the case of activating as well as inactivating mutations of the CaSR, and it is notable that renal hypophosphaturia occurred in homozygous but not heterozygous *Nuf* mice consistent with the idea that the CaSR normally suppresses renal phosphate excretion including PTH-induced inhibition of phosphate reabsorption (Riccardi et al., 2000; Ba et al., 2003; reviews: Riccardi and Valenti, 2016) and thus promotes phosphate retention. The disorder is amenable to treatment with negative modulators of the CaSR, also known as calcilytics (see below; Mayr et al., 2016; Nemeth and Goodman, 2016).

#### Disorders of calcium metabolism arising from autoantibodies that target the CaSR

In addition to the impact of inactivating or activating CaSR mutations on calcium metabolism and parathyroid function as described above, several studies have drawn attention to the clinical impact of autoantibodies that target the CaSR with either inactivating (Kifor et al., 2003; Pallais et al., 2004) or activating (review: Brown, 2009) effects, presumably dependent on the peptide epitope that is recognized. These autoimmune disorders of calcium metabolism resemble other autoimmune endocrinopathies such as Grave's disease (review: Thakker, 2004). In one of these disorders associated with autoimmune polyendocrinopathy, autoantibodies to several CaSR epitopes have been identified corresponding to residues 41–69 at the receptor's N-terminus, 114–126 at the dimer interface, and 171–195 in the vicinity of the Venus FlyTrap (VFT) domain's binding cleft (Kemp et al., 2010).

### Transgenic mouse models—impact of inactivating CaSR mutations on parathyroid function

The first reported transgenic mouse in which the CaSR was “knocked out,” was homozygous for a 20 bp insertion that disabled incorporation of CaSR exon-5 (referred to as CaSR exon-4 in the paper) into the mature, fully processed mRNA (Ho et al., 1995). CaSR exon-5 encodes residues 465–536 (http://www.casrdb.mcgill.ca) at the extreme C-terminal end of the VFT domain, immediately prior to the start of the Cysteine-rich domain. Mice with this genotype exhibited a condition comparable to NSHPT in which homozygotes were normal at birth but exhibited severe growth retardation and markedly reduced muscle power in the days after birth (Ho et al., 1995).

The results of biochemical analyses demonstrated the cardinal features of primary hyperparathyroidism including markedly elevated plasma Ca^2+^ concentration, suppressed plasma inorganic phosphate concentration, and markedly elevated serum PTH levels. In addition, the parathyroid glands were enlarged with prominent chief cell hyperplasia (Ho et al., 1995). These findings are consistent with a severe resistance syndrome arising from markedly impaired Ca^2+^-dependent feedback control of PTH secretion i.e., with loss of the parathyroid Ca^2+^ sensor.

Heterozygotes, unlike the homozygotes, were phenotypically normal in the weeks and months after birth but exhibited mild biochemical disturbances consistent with FHH in humans including mildly elevated plasma Ca^2+^ concentration, suppressed renal calcium excretion, and inappropriately normal plasma PTH levels. These findings suggest a mildly impaired but intact parathyroid Ca^2+^ sensing mechanism together with impaired extracellular Ca^2+^-dependent inhibition of renal Ca^2+^ reabsorption resulting in an increase in the setpoint of the calciostat.

### Is the parathyroid equipped with an alternative calcium-sensing receptor?

While other class C GPCRs, like the CaSR, exhibit Ca^2+^-sensing properties (Kubo et al., 1998; Wise et al., 1999; Christiansen et al., 2007) it seems unlikely that the parathyroid is equipped with an alternative CaSR since, as described above, mice that are homozygous for either global (Ho et al., 1995) or tissue-selective (Chang et al., 2008) knockouts of the CaSR exhibit a severe, uncompensated form of primary hyperparathyroidism in which the plasma levels of both PTH and calcium are markedly elevated from birth. The phenotype suggests a marked impairment of Ca^2+^_o_-dependent negative feedback on PTH secretion with attendant hyperparathyroidism and PTH-dependent bone resorption. Thus, if the parathyroid expresses an alternative or supplementary calcium sensor, it is unable to compensate for loss of the CaSR. It is possible that under some circumstances Ca^2+^-sensing is mediated not by CaSR homodimers but by CaSR heterodimers involving other members of GPCR family C including metabotropic glutamate receptors or GABA_B1_ receptors as noted above (Gama et al., 2001; Chang et al., 2007; Cheng et al., 2007).

Previous work suggested a role for Ca^2+^-permeable channels in the control of PTH secretion based on observations that stereoisomers of the Ca^2+^ channel modulator 202–791 either inhibited (+202 to 791) or stimulated (−202 to 791) PTH secretion (Fitzpatrick et al., 1986b), and antibodies that target skeletal muscle Ca^2+^ channels also modulated PTH secretion (Fitzpatrick et al., 1988). Other Ca^2+^ channel activators, including maitotoxin (Fitzpatrick et al., 1989), and the diltiazem analog TA-3090 (Chen and Brown, 1990) were also found to inhibit PTH secretion. This work was “turned on its head” by the successful development of “calcimimetics” by structural modification of an L-type Ca^2+^ channel blocker, fendiline (Nemeth et al., 1998), and the subsequent demonstration that modulation of PTH secretion by these agents arises not from actions on Ca^2+^ channels but rather the cloned CaSR (Nemeth et al., 2004; review: Nemeth, 2006). Thus, various agents that modulate Ca^2+^ channel activity can also interact with an allosteric site in the CaSR's heptahelical domain (Leach et al., 2016). Calcimimetics, positive modulators of the CaSR, and calcilytics, negative modulators of the CaSR, are discussed in greater detail below.

Nevertheless, more recent work raises the possibility that Ca^2+^-permeable channels may indeed contribute to the control of PTH secretion. Thus, parathyroid cells express NMDA receptor subunits and NMDA inhibits PTH secretion (Parisi et al., 2009). While these receptors may contribute to the tonic control of PTH secretion, it is not known whether Ca^2+^ fluxes arising from the activation of NMDA receptors are sensitive to Ca^2+^_o_ concentration in parathyroid cells. In addition, various amino acids and amino acid analogs are known to interact with the CaSR (Conigrave et al., 2000b, 2004; review: Conigrave and Hampson, 2010) and it is not yet clear whether the inhibitory effect of NMDA on PTH secretion is exerted by the activation of Ca^2+^-permeable ion channels or via positive modulation of the CaSR.

## The present

### Development of calcimimetics and their utility in several forms of hyperparathyroidism

As noted above, calcimimetics were developed from the Ca^2+^ channel blocker fendiline that induces Ca^2+^_i_ mobilization and suppresses PTH secretion from bovine parathyroid cells (Nemeth et al., 1998; review: Nemeth, 2006). Drug development resulted in a new class of pharmaceuticals, the phenylalkylamine calcimimetics, which are positive allosteric modulators of the CaSR that markedly enhance the sensitivity of CaSR-mediated intracellular signaling pathways to Ca^2+^_o_ (Nemeth et al., 1998). Early examples included NPS R467 and NPS R568, which together with their less potent S-isomers have been key agents for the analysis of CaSR-mediated effects in various cell and tissue systems. More recent examples include cinacalcet, an agent that is well-absorbed orally (Nemeth et al., 2004) and is effective clinically in the treatment of both secondary hyperparathyroidism due to chronic kidney disease (Moe et al., 2005; Messa et al., 2008) as well as primary hyperparathyroidism (Peacock et al., 2005, 2011; see also review: Nemeth and Shoback, 2013).

One key effect of calcimimetics is suppression of the serum PTH level. In primary hyperparathyroidism, for example, in which the plasma total calcium concentration is typically elevated from its normal upper limit of 2.6 mM to around 2.8–3.0 mM, oral therapy with cinacalcet suppressed serum PTH levels and restored the plasma calcium concentration into the normal range for up to 12 months or more (Peacock et al., 2005). Another key effect is suppression or even reversal of parathyroid hyperplasia. For example, cinacalcet suppresses parathyroid cell proliferation and reduces gland size in models of primary (Imanishi et al., 2011) and secondary (Colloton et al., 2005; Miller et al., 2012) hyperparathyroidism, and also induces apoptosis in second hyperparathyroidism (Tatsumi et al., 2013).

The demonstration that calcimimetics from the same class and across different classes exhibit different biased signaling profiles (Davey et al., 2012) is encouraging efforts to develop new generation calcimimetics in support of tissue-specific CaSR-targeted pharmacotherapy e.g., parathyroid vs. kidney vs. thyroid C-cells (review: Leach et al., 2015). Recent modeling of calcimimetic binding in the CaSR's heptahelical domain suggests that agents such as A265347 with less pronounced biased signaling profiles may bind more deeply in the allosteric pocket (Leach et al., 2016).

More recently, a peptide activator of the CaSR (AMG-416; L-Cys-AcDCys-DAla-(DArg)_2_-DAla-DArgNH_2_) has entered clinical practice for the treatment of patients with secondary hyperparathyroidism on hemodialysis (Bell et al., 2015). Administered intravenously it has superior pharmacokinetics including effective suppression of PTH levels beyond 24 h (Walter et al., 2013) due, presumably, to its ability to form a di-sulfide with CaSR residue C482 in its extracellular domain (Alexander et al., 2015).

### Calcilytics

Several classes of calcilytics (negative modulators of the CaSR) have been developed. These agents, in general, bind in the HH domain and suppress CaSR signaling. For this reason, they have proved useful in assessing the role of the CaSR in Ca^2+^- or L-amino acid-induced cellular or tissue responses (e.g., Dvorak et al., 2004; Daly et al., 2013). In the parathyroid, calcilytics promote PTH secretion by reversing the inhibitory action of the CaSR (Nemeth et al., 2001). As a consequence, it was hoped that these agents might prove useful in the treatment of osteoporosis by elevating serum PTH levels to emulate the action of intermittent subcutaneous injections of PTH1–34 (teriparatide). However, none of the calcilytics that have entered human clinical trials, thus far, have been successful in significantly increasing bone density or reducing fracture risk (review: Nemeth and Goodman, 2016). Two main explanations seem reasonable: (i) the maximum increase in the serum level of endogenous PTH is significantly less than that achieved by subcutaneous injections of PTH1–34 (e.g., Kimura et al., 2011); or (ii) calcilytics suppress CaSRs in cells of the osteoblast lineage to interfere with PTH-induced cell maturation and key differentiated functions including matrix synthesis and mineralization (Dvorak et al., 2004).

### Nutrient activators of the CaSR

In addition to its regulation by Ca^2+^ ions, the CaSR also responds promiscuously to L-amino acids of various classes (Conigrave et al., 2000b), and one of the most potent, L-Trp, has been shown recently to bind in the receptor's VFT domain ligand-binding groove (Geng et al., 2016; see below). This behavior resembles that of several class C GPCRs (Conigrave and Hampson, 2006, 2010) and supports macronutrient sensing in various tissues including the gastrointestinal tract (review: Conigrave and Brown, 2006). Based on the signaling pathway analysis performed to date, however, Ca^2+^_o_ and L-amino acids are not equivalent activators. In particular, L-amino acids preferentially activate a Ca^2+^_i_ mobilizing pathway and have more limited actions on PI-PLC and ERK_1/2_ (review: Conigrave and Ward, 2013). Nevertheless, L-amino acids are potent activators of Ca^2+^_i_ mobilization in parathyroid cells and also suppress PTH secretion at physiologically relevant concentrations (Conigrave et al., 2004). Furthermore, glutathione and various analogs (e.g., S-methylglutathione) also activate Ca^2+^_i_ mobilization and suppress PTH secretion, presumably by binding to the same VFT domain ligand-binding groove (Broadhead et al., 2011). These findings imply that protein nutritional state is negatively coupled to the control of PTH secretion and thus serum PTH levels. The full significance of these effects, however, is not yet known (see below).

### Control of CaSR gene expression

Analysis of the promoter regions of the CaSR gene has led to the identification of two key positive modulators of expression: (i) inflammatory cytokines including IL-1β, IL-6 and TNFα (Canaff and Hendy, 2005); and (ii) hormonally active analogs of vitamin D including 1,25-dihydroxyvitamin D_3_ (Canaff and Hendy, 2002), and possibly 25-hydroxyvitamin D_3_, whose plasma levels are nearly 1000-fold higher. These results suggest that CaSR expression may be upregulated in the parathyroid and other CaSR-expressing tissues in response to various inflammatory conditions and in response to elevations in either serum 1,25-dihydroxyvitamin D_3_ or 25-hydroxyvitamin D_3_ levels.

## Recent developments and the future

### G-protein coupling

The CaSR couples to various G-proteins (review: Conigrave and Ward, 2013). Notable from the perspective of parathyroid function are G_i_, which suppresses agonist-stimulated GPCR-mediated cAMP production and contributes to the activation of ERK_1/2_ at least in part via β-arrestin, and G_q/11_, which activates PI-PLC and induces Ca^2+^_i_ mobilization, with attendant activation of several protein kinase C isoforms and ERK_1/2_.

Both the G_i_ and G_q/11_ pathways appear to be important for the inhibitory control of PTH secretion. With respect to G_q_ and G_11_, it is now known that Gα_q_ and Gα_11_ are required for the normal control of PTH secretion. Thus, in a transgenic mouse in which parathyroid-specific ablation of Gα_q_ was produced on a global Gα_11_ null background, severe neonatal hyperparathyroidism was observed (Wettschureck et al., 2007) and resembled the phenotypes of both global (Ho et al., 1995) and parathyroid-specific (Chang et al., 2008) ablation of the CaSR. These findings demonstrate that G_q_ and G_11_ are required for CaSR-mediated control of PTH secretion and thus lie at the top of a key inhibitory signaling pathway(s). Consistent with these findings, inactivating and activating mutations of the human Gα_11_ gene have been shown respectively to underlie variant forms of FHH (FHH2) and ADH (ADH2; Nesbit et al., 2013a; Gorvin et al., 2016; Piret et al., 2016).

Under certain circumstances, the CaSR also couples to G_s_ (review: Conigrave and Ward, 2013) but the significance of this pathway for the control of PTH secretion is unknown. It is interesting to speculate that the “inactive” form of the receptor, which is promoted under conditions of low Ca^2+^ and high phosphate concentrations (Geng et al., 2016) might preferentially couple to G_s_ in the parathyroid.

### Receptor trafficking

Receptor trafficking studies have largely focused on cell systems in which the CaSR is expressed heterologously (reviews: Breitwieser, 2013, 2014). These studies demonstrate that trafficking of the CaSR is modulated by various binding partner proteins (review: Huang and Miller, 2007), can be promoted by allosteric modulators such as cinacalcet and NPS-2143 acting as pharmaco-chaperones (Leach et al., 2013), and is sensitive to receptor-dependent signaling (Grant et al., 2011, 2012; review: Breitwieser, 2012). In the parathyroid, the CaSR interacts with caveolin and is thus likely to localize to sub-domains of the plasma membrane known as caveolae (Kifor et al., 1998). In addition, recent findings suggest that the CaSR is processed between the plasma membrane and intracellular endosomes via clathrin-coated vesicles since mutations of Arg15 of the sigma (σ) subunit of the clathrin-binding protein AP2 have been linked to a variant form of FHH, now known as FHH3 (Nesbit et al., 2013b). The findings suggest that the formation, and/or maintenance, of CaSR signaling complexes is impaired under conditions in which clathrin-coated vesicle-mediated processing of the CaSR is impaired.

### X-ray crystal structures

While X-ray crystal structures of class C GPCR VFT domains (Kunishima et al., 2000; Tsuchiya et al., 2002), entire extracellular (VFT-plus-Cys-rich) domains (Muto et al., 2007), and even heptahelical domains (Doré et al., 2014) have been reported over the last 15 years, crystal structures for CaSR domains have only recently become available (Geng et al., 2016; Zhang et al., 2016).

These newly described CaSR structures provide information on the inactive and active forms of its VFT domain (Geng et al., 2016; Zhang et al., 2016) and entire extracellular domain (Geng et al., 2016). While the protein conformations of the active forms of the VFT domain structures were almost identical, the identification of divalent cation, and anion binding sites were quite different in the structures reported by the two groups. Zhang et al. (2016) identified just one Ca^2+^ site in the active form of the VFT domain and relied on modeling of electron densities to ascribe it to the ligand-binding cleft, where it was closely associated with an L-amino acid-binding site. Surprisingly, however, they identified a formaldehyde derivative rather than the native form of L-Trp in the site.

In the structures described by Geng et al. (2016), on the other hand, an anomalous mapping strategy was used to identify four, previously unrecognized, Ca^2+^ binding sites, one of which (“Site 2”) was present in both the inactive and active structures and three of which were only identified in the active structure and, thus, may act to stabilize it. Interestingly, no Ca^2+^ binding site was located in the closed (active) form of the agonist-binding cleft in the structure reported by Geng et al., which was occupied instead by the amino acid L-Trp (Geng et al., 2016). In addition, Geng et al. identified several binding sites for inorganic phosphate in the inactive structure (Geng et al., 2016), raising the possibility that not only the Ca^2+^_o_ concentration but also the ratio of Ca^2+^_o_ to phosphate concentrations may control the receptor's transition between inactive and active states.

The findings that the receptor binds inorganic phosphate (P_i_) as well as Ca^2+^ ions and that Ca^2+^ stabilizes the active state, whereas P_i_ stabilizes the inactive state have potentially important implications for understanding parathyroid function since elevated P_i_ concentrations stimulate PTH secretion (Slatopolsky et al., 1996) whereas elevated Ca^2+^_o_ inhibits it. Does the CaSR modulate its response to Ca^2+^_o_ according to the background level of inorganic phosphate? Does the Ca:P_i_ ratio determine PTH secretion rates by controlling the activation state of the CaSR? Does the CaSR act as a phosphate sensor in other tissues such as osteocytes or osteoblasts in bone?

### Unresolved problems

There are several unresolved problems. Four of them are considered below in the form of sets of questions.

#### Question-set 1

What drives intrinsic PTH secretion and how does the CaSR suppress it in a G_i_-independent manner? Is spontaneous PTH secretion truly constitutive, implying that the pathway by which PTH vesicles undergo exocytosis is unregulated? Alternatively, is it promoted by receptors expressed on the surface of parathyroid cells that are either constitutively active or exposed to locally released activators such as histamine from mast cells or prostanoids from chief or oxyphil cells?

#### Question-set 2

What is the significance of amino acid-binding to the CaSR (Geng et al., 2016) for parathyroid function? Does the parathyroid CaSR read the local concentrations of L-amino acids arising from export of amino acids from the cytoplasm or are they determined by the amino acid concentrations in the bulk plasma. Does amino acid sensing by the CaSR primarily affect PTH secretion under conditions of protein deficiency and reductions in plasma amino acid levels as suggested by the phenomenon of secondary hyperparathyroidism in subjects on low protein diets (reviews: Conigrave et al., 2002, 2008) or does it act primarily to suppress PTH secretion under conditions of protein excess as suggested by parathyroid cell responses *in vitro* (Conigrave et al., 2004). Alternatively, might L-amino acid sensing by the CaSR provide a mechanism for adjusting the inhibitory gain on the receptor to the level of amino acid-dependent PTH synthesis?

#### Question-set 3

What is the significance of CaSR heterodimerization for parathyroid function? Is the parathyroid subject solely to control by CaSR homodimers or are some Ca^2+^-dependent signaling pathways (e.g., for the control of parathyroid chief cell number, or PreProPTH gene expression) subject to control by CaSR heterodimers with metabotropic glutamate receptors (Gama et al., 2001) or GABA_B1_ receptors (Chang et al., 2007)?

#### Question-set 4

Can CaSR expression be effectively upregulated in hypercalcemic conditions such as primary hyperparathyroidism or FHH to restore physiological control of plasma calcium levels and Ca^2+^_o_-dependent suppression of PTH secretion? Can CaSR expression be effectively downregulated in hypocalcemic conditions such as ADH to restore physiological control of plasma calcium and PTH levels? Can tissue-selective modulators of the vitamin D receptor or cytokine receptors, or other strategies, be developed for the control of parathyroid CaSR expression?

## Concluding remarks

The role of the parathyroid in the whole body calcium economy is so important that the negative feedback loop by which PTH elevates plasma Ca^2+^ and Ca^2+^, in turn, suppresses PTH secretion largely defines its place in human biology. Expression cloning of the CaSR, its identification as the key Ca^2+^ sensor of the parathyroid, and evaluation of its roles in normal tissue biology and in human disease have resolved key issues in calcium metabolism. New paradigms of Ca^2+^-mediated control of tissue function and of the CaSR in macronutrient-sensing have followed. Incredibly, the molecular mechanism by which the CaSR suppresses PTH secretion is only partially solved: for the situation in which PTH secretion is stimulated by neurotransmitters or hormones that elevate cAMP levels. The mechanisms by which the CaSR suppresses intrinsic PTH secretion or the secretion of PTH downstream of hormones that activate PTH secretion by non-cAMP pathways remain undefined. Newly available X-ray crystal structures for the CaSR extracellular domain in its inactive and active conformations provide new opportunities to investigate the Ca^2+^ sensing mechanism.

## Author contributions

The author confirms being the sole contributor of this work and approved it for publication.

## Funding

The author's work on the role of the calcium-sensing receptor has been funded by the National Health & Medical Research Council of Australia (project grants APP1011922, APP1026962, and APP1085143).

### Conflict of interest statement

The author declares that the research was conducted in the absence of any commercial or financial relationships that could be construed as a potential conflict of interest.
